# Rare GNAO1 Variant Presenting with Deep Brain Stimulation‐Responsive Jaw‐Opening Dystonia

**DOI:** 10.1002/mdc3.70048

**Published:** 2025-03-22

**Authors:** Fabian Maass, Saskia Biskup, Vesna Malinova, Christiane Weinrich, Christoph van Riesen

**Affiliations:** ^1^ Department of Neurology University Medical Center Göttingen Göttingen Germany; ^2^ CeGaT, Center for Genomics and Transcriptomics Tübingen Germany; ^3^ Department of Neurosurgery University Medical Center Göttingen Göttingen Germany; ^4^ German Center for Neurodegenerative Diseases (DZNE) Göttingen Germany

**Keywords:** DBS, GNAO1, jaw‐opening dystonia

The GNAO1 gene encodes the α subunit of the heterotrimeric guanine nucleotide‐binding protein (G protein). Pathogenic variants have been linked to the severe phenotype of autosomal‐dominant developmental and epileptic encephalopathy 17 (DEE17, Online Mendelian Inheritance in Man [OMIM] 615473) and to neurodevelopmental disorder with involuntary movements (NEDIM, OMIM 617493).^1^ The movement disorders associated with this condition are primarily characterized by dystonia and choreoathetosis, which may be persistent or paroxysmal in nature. A distinct risk of spontaneous or triggered exacerbation exists, which can lead to life‐threatening hyperkinetic crises (see Briere et al^1^ for further details). A prominent upper‐body dystonia with slow progression, which may be associated with dysarthria, without severe encephalopathy has also been recently characterized.^2^ Here, we present a rare phenotype of severe jaw‐opening dystonia associated with a pathogenic GNAO1 variant, demonstrating responsiveness to globus pallidus internus (GPi)‐deep brain stimulation (DBS).

A 21‐year‐old Caucasian woman, accompanied by her mother, was referred to our specialized outpatient clinic for movement disorders with an oromandibular dystonia of unknown etiology. The patient reported that the initial symptoms manifested during her early childhood at nursery school, where speech‐language pathologists treated speech difficulties. The subject's mother exhibited dysarthric speech patterns, although she did not present with oromandibular dystonia. Apart from that, there was no definitive family history. At the age of 14, she began to show symptoms of progressive oromandibular dystonia, including tongue deviation and a jaw‐opening pattern. Despite treatment with levodopa and trihexyphenidyl, the symptoms exhibited only minimal improvement. At the age of 17, the patient was referred to the outpatient clinic for phoniatrics at our hospital. Endoscopic diagnostics revealed no pathological conditions concerning the oral cavity, the pharynx, and the larynx. Additionally, an initial observation of a deviation of the tongue to the right side was noted, without any indications of paralysis. The voice was described to be hoarse and deep, with slowed speech and minor aspects of velopharyngeal insufficiency. Under stressful conditions and after longer periods of speech stress, dystonic movements in the orbicularis oris muscle were identified. The administration of botulinum toxin did not result in a notable improvement and, as a consequence, was terminated. The dystonia exhibited further progression, necessitating the application of pressure with a hand to facilitate mouth closure throughout the day, potentially reflecting a geste antagoniste.

The clinical examination (Video 1) revealed the presence of jaw‐opening dystonia, tongue deviation to the right, a dysarthric speech pattern, and a syndactyly of the right toe. She was unable to move her tongue to the left voluntarily. In contrast to the phoniatric examination carried out several years ago, a distinct impairment in tongue mobility had now become evident. There was no atrophy of the tongue muscles and no positive signs indicating a functional disorder, therefore, the tongue deviation was interpreted as a sustained dystonic posture. Genetic testing of the patient revealed the presence of a pathogenic variant (c.617>A;p.Arg206Gln) in the GNAO1 gene, the mother declined to undergo testing. This variant has previously been described by Wirth and colleagues^2^ in a patient with symptom onset at the age of 15 years, presenting with segmental oromandibular and cervical dystonia and additional dysarthria. Although our patient also suffered from oromandibular dystonia, the presentation is markedly different as our patient has prominent jaw opening dystonia. This observation gives rise to the hypothesis that the manifestation of this observed variance may be attributed to the influence of modifying genes, variability in gene expression or epigenetic factors as has been found in other dystonia genes.^3^ In the case described by Wirth et al,^2^ there was no response to levodopa and tetrabenazine, and it is noteworthy that there was a mild worsening by GPi‐DBS. A magnetic resonance imaging scan showed no obvious morphological changes (Fig. S1) and an electroencephalogram showed no evidence of epileptic encephalopathy. Neuropsychological assessment revealed only minor cognitive abnormalities on the Mini‐Mental State Examination (28/30 points) and no depression (Beck's Depression Inventory‐II 1 point). Given the severity of the symptoms, implantation of DBS into the posteroventral pallidum was performed (Data S1). Lead placement was visualized by postoperative imaging using Brainlab software (see Fig. 1). After adjusting the final stimulation settings (Case +, 1%–100%; 3.2 mA, 90 μs, 130 Hz), a sustained improvement, primarily in jaw‐opening dystonia (Videos 2 and 3), led to a significant enhancement in speech. The available literature provides limited information regarding optimal DBS settings for oromandibular dystonia. However, the parameters used in this study are consistent with those reported in the existing literature to be beneficial.^4^, ^5^

**Video 1:** Preoperative video illustrating the presence of severe jaw opening dystonia and right‐sided tongue deviation. The patient states that she is unable to close her mouth without using her hand for assistance. She also stated that she was completely unable to move her tongue to the left voluntarily. A geste antagoniste is used to achieve a closed mouth position.

**FIG. 1:**
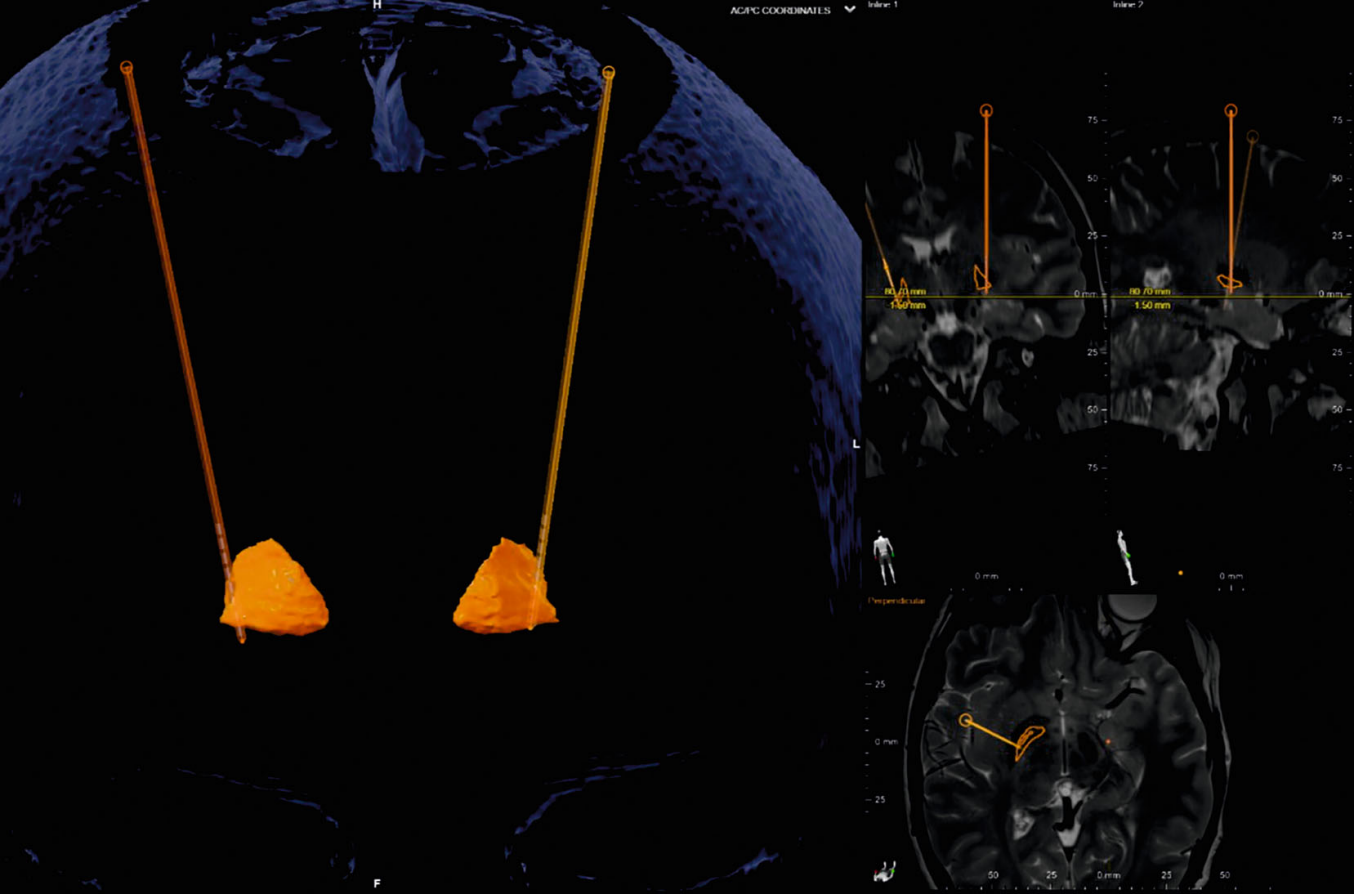
Visualization of electrode placement in the posteroventral globus pallidus internus (GPi) in the Brainlab software.

**Video 2:** Postoperative video showing the clinical effect of pallidal deep brain stimulation on the jaw‐opening dystonia. The patient is now able to close her mouth spontaneously and speak freely which is primarily attributable to the alleviation of the jaw‐opening dystonia, rather than an improvement in the tongue dystonia.

**Video 3:** Postoperative video showing gait examination. Consistent with the pre‐deep brain stimulation assessment, there are no signs of dystonic gait or limb dystonia.

The case described has important clinical implications. Here, we report a very rare variant in the GNAO1 gene that has only been described once before in the literature.^2^ Interestingly, given the large phenotypic spectrum of GNAO1‐associated disorders, a very similar phenotype with oromandibular dystonia and dysarthria was reported in this case. It is noteworthy that a mild worsening was observed in the aforementioned case following GPi‐DBS, which contrasts with the positive response observed in our case. A recent meta‐analysis reported that pallidal DBS is generally effective and safe in GNAO1‐associated dystonia.^6^ This has also been reported concerning the mild dystonic, non‐encephalopathic phenotype.^2^ Although oromandibular dystonia appears to be a common feature of GNAO1‐associated dystonia, a predominant and severe jaw‐opening dystonia subtype has not been explicitly identified, thereby broadening the clinical spectrum of GNAO1‐associated dystonia. The impact of GPi‐DBS on jaw‐opening dystonia has only been demonstrated on a limited number of occasions. Our case study provides evidence to suggest that GPi‐DBS may be a potential treatment option, when other therapies have been unsuccessful.

## Author Roles

(1) Research Project: A. Conception, B. Organization, C. Execution; (2) Statistical Analysis: A. Design, B. Execution, C. Review and Critique; (3) Manuscript Preparation: A. Writing of the First Draft, B. Review and Critique;

F.M.: 1A, 1B, 1C, 3A, 3B

S.B.: 1B 1C, 3B

V.M.: 1B, 1C, 3B

M.B.: 1B, 3B

C.v.R.: 1A, 1B, 1C, 3A, 3B

## Disclosures

**Ethical Compliance Statement**: The authors confirm that the approval of an institutional review board was not required for this work. We confirm that patient consent has been sought and allowed for this case and its publication. We confirm that we have read the Journal's position on issues involved in ethical publication and affirm that this work is consistent with those guidelines.

**Funding Sources and Conflicts of Interest**: No specific funding was received for this work. The authors declare that there are no conflicts of interest relevant to this work.

**Financial Disclosures for the Previous 12 Months**: FM and CvR received speaker honoraria from Abbvie and travel grants from Bial.

## Supporting information


**Figure S1.** MRI imaging (T1 sequences), exemplified in three different regions: periventricular (A), basal ganglia (B), and lower brainstem (C), showing no evidence of encephalopathy.

**Data S1.** Supplement (DBS details): includes further details concerning the DBS system.

## Data Availability

Data sharing is not applicable to this article as no new data were created or analyzed in this study.
