# Supplementary figures and images for: Rare GNAO1 Variant Presenting with Deep Brain Stimulation‐Responsive Jaw‐Opening Dystonia
Source: Mov Disord Clin Pract. 2025 Mar 22;12(8):1196–9. doi: 10.1002/mdc3.70048 (PMC12371467; doi:10.1002/mdc3.70048)

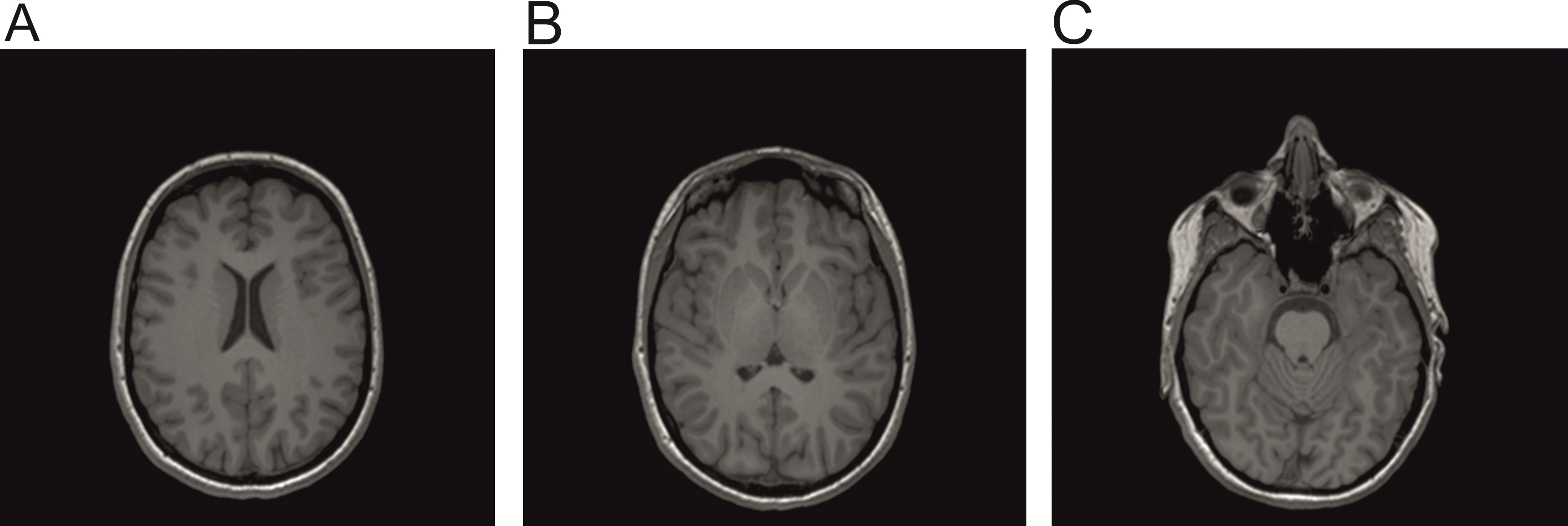

Supplement: Supplementary file 1 — Figure S1. MRI imaging (T1 sequences), exemplified in three different regions: periventricular (A), basal ganglia (B), and lower brainstem (C), showing no evidence of encephalopathy. [file MDC3-12-1196-s002.tif]
