# Supplementary material for: Rare GNAO1 Variant Presenting with Deep Brain Stimulation‐Responsive Jaw‐Opening Dystonia
Source: Mov Disord Clin Pract. 2025 Mar 22;12(8):1196–9. doi: 10.1002/mdc3.70048 (PMC12371467; doi:10.1002/mdc3.70048)
Supplement: Supplementary file 2 — Data S1. Supplement (DBS details): includes further details concerning the DBS system. [file MDC3-12-1196-s001.docx]

**Rare GNAO1 variant presenting with DBS-responsive jaw-opening dystonia**

**(supplement – DBS details)**

Fabian Maass, MD^1^, Saskia Biskup, MD, PhD^2^, Vesna Malinova, MD^3^, Christiane Weinrich, MD^1^, Christoph van Riesen, MD^1,4^

^1^ University Medical Center Göttingen, Germany, Dept. of Neurology

^2^ CeGaT, Center for Genomics and Transcriptomics, Tübingen, Germany

^3^ University Medical Center Göttingen, Germany, Dept. of Neurosurgery

^4^ Germany Center for Neurodegenerative Diseases (DZNE), Göttingen, Germany

DBS description:

Given the severity of the symptoms, frame-based stereotactic implantation of DBS electrodes (DB 2201-30AC; Boston Scientific, Marlborough, MA) into the posteroventral pallidum was performed with microelectrode recording and macrostimulation to assess side effects. The electrodes were connected to an implantable rechargeable neural stimulator (Boston Scientific Vercise Gevia). After adjusting the final stimulation settings (Case +, 1- 100%; 3.2 mA, 90µs, 130 Hz), a significant improvement of the oromandibular dystonia could be demonstrated (Video 2 and Video 3), with the ability to speak freely for several hours (examined four weeks post-surgery).
